# Investigation of the Associations of Novel Inflammatory Biomarkers—Systemic Inflammatory Index (SII) and Systemic Inflammatory Response Index (SIRI)—With the Severity of Coronary Artery Disease and Acute Coronary Syndrome Occurrence

**DOI:** 10.3390/ijms23179553

**Published:** 2022-08-23

**Authors:** Ewelina A. Dziedzic, Jakub S. Gąsior, Agnieszka Tuzimek, Justyna Paleczny, Adam Junka, Marek Dąbrowski, Piotr Jankowski

**Affiliations:** 1 Medical Faculty, Lazarski University in Warsaw, 02-662 Warsaw, Poland; 2 Department of Internal Medicine and Geriatric Cardiology, Centre of Postgraduate Medical Education, 01-813 Warsaw, Poland; 3 Department of Pediatric Cardiology and General Pediatrics, Medical University of Warsaw, 02-091 Warsaw, Poland; 4 Department of Pharmaceutical Microbiology and Parasitology, Faculty of Pharmacy, Wroclaw Medical University, 50-556 Wroclaw, Poland; 5 Department of Cardiology, Bielanski Hospital, 01-809 Warsaw, Poland; 6 Department of Epidemiology and Health Promotion, School of Public Health, Center of Postgraduate Medical Education, 01-826 Warszawa, Poland

**Keywords:** systemic inflammatory index, systemic inflammatory response index, coronary artery disease, coronary angiography, acute coronary syndrome

## Abstract

Atherosclerosis, the underlying cause of coronary artery disease (CAD), has a significant inflammatory component. White blood cell count is an affordable and accessible way to assess the systemic immune response, as it comprises many subgroups with distinct and complex functions. Considering their multidirectional effect on atherosclerosis, new biomarkers integrating various leukocyte subgroups, the Systemic Inflammatory Index (SII) and the Systemic Inflammatory Response Index (SIRI), were recently devised to describe the balance between inflammation and immune reaction. This research aimed to evaluate the relationship of the intensity of inflammation measured by these biomarkers with the severity of CAD assessed with coronary angiography and with the diagnosis of acute coronary syndrome (ACS) or stable CAD in 699 patients. SIRI, but not SII, was associated with the diagnosis, having the highest values for patients with ACS (STEMI), significantly higher than in patients with stable CAD (*p* < 0.01). The highest SII and SIRI values were observed in patients with three-vessel CAD. SII and SIRI require further in-depth and well-designed research to evaluate their potential in a clinical setting.

## 1. Introduction

Data collected by the World Health Organization (WHO) indicate that cardiovascular diseases (CVD) are the leading cause of death in the world. In 2019, the worldwide mortality from acute coronary syndrome (ACS) exceeded 15 million, and 40% of them were premature deaths in patients under 70 years of age [1].

The main cause of CVD is atherosclerosis, a chronic, systemic, inflammatory disease of the walls of the large arteries with lipid accumulation in the matrix of intima [2]. The immune system plays a significant role in every step of atherogenesis, from endothelial injury to plaque rupture with clot formation [3,4,5]. Imbalance of pro- and anti-atherogenic immune mechanisms leads to clinical manifestations of atherosclerosis, e.g., coronary artery disease (CAD), myocardial infarctions (MI), or strokes [6,7].

Considering the complex pathophysiological relationship between atherosclerosis and CAD [8], the association of inflammatory markers with these diseases deserves a thorough investigation. Elevated serum C-reactive protein (CRP) was previously correlated with a higher risk of CVD [9,10]. Other affordable and commonly available hematologic markers based on white blood cell count and their subtypes (neutrophils, lymphocytes, and monocytes) were associated with a risk of cardiovascular complications such as MI and stroke [11,12,13,14], as well as overall mortality for any reason [15,16]. The monocyte–lymphocyte ratio (MLR), neutrophil–lymphocyte ratio (NLR), and platelet–lymphocyte ratio (PLR) are biomarkers that represent a wide diversity of immune pathways and cell functions. As they bring together the impact of two different cell lines that influence each other, their overall CVD and mortality predictive value increases synergistically [17,18,19].

Novel inflammatory biomarkers, systemic inflammatory index (SII), and systemic inflammatory response index (SIRI), have recently been described. They were found to predict a poor prognosis more precisely in patients with colorectal and esophageal cancer compared to NLR and PLR [20,21]. SII employs three blood cell subtypes (neutrophils, lymphocytes, and platelets), reflecting the balance between inflammation and the immune response to it [22,23]. In cardiac patients, elevated SII was associated with an increased risk of CAD and its greater severity [24,25,26], as well as a worse development of collateral circulation in heart muscle [27] or a higher risk of major adverse cardiovascular events (MACE) in patients with heart failure [28] after coronary intervention [29,30] or cardiosurgery [31,32,33,34]. SII was also described as an independent predictor of massive pulmonary embolism [35], a contrast-induced nephropathy risk factor in patients undergoing coronary angiography [36,37], a risk factor for postoperative and recurrent atrial fibrillation after cardiac surgery [38], as well as for developing patent systolic dysfunction in postpartum cardiomyopathy [39].

SIRI comprises the absolute number of neutrophils, monocytes, and lymphocytes [40]. In a 10-year observation, SIRI was found to have an association with CVD occurrence [41] and an increased risk of supraventricular tachycardia in patients with a history of stroke [42]. In patients who underwent percutaneous coronary intervention (PCI) due to ACS, SIRI was an independent predictor of MACE [29,43].

This research aims to evaluate the relationship of the intensity of inflammation measured by new biomarkers—SII and SIRI—with the severity of CAD and the diagnosis of ACS or stable CAD in patients who underwent coronary angiography.

## 2. Results

### 2.1. Characteristics of Participants

Details on the study group are presented in Table 1.

### 2.2. Association between Measured and Derived Parameters and Severity of Cad

**Figure 1:**
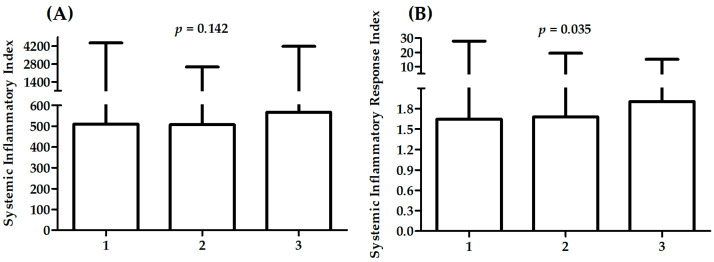
Differences in SII (**A**) and SIRI (**B**) between patients with different CASSC.

**Figure 2:**
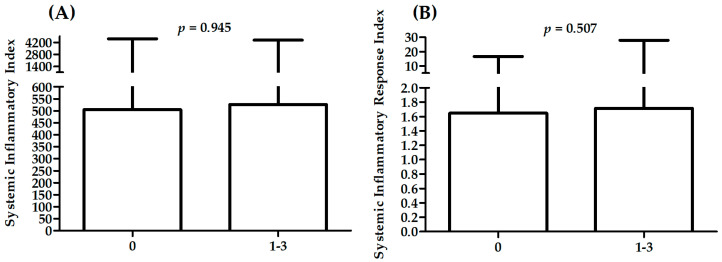
Differences in SII (**A**) and SIRI (**B**) between patients with CASSC 0 and CASSC 1–3.

Table 2 presents a comparison between patients with different CASSC. A significant difference in sex distribution was observed between CASSC groups. There was also a significant difference in distribution of patients with different cause of hospitalization (STEMI: ST-elevation myocardial infarction; NSTEMI: non-ST-elevation myocardial infarction; UA: unstable angina), history of previous MI, hypertension, and smoking status. Patients with CASSC 3 were significantly older than others (*p* < 0.05 for all comparisons, i.e., vs. patients with CASSC 0, 1, and 2), presented the lowest values of HDL (*p* < 0.01 for all comparisons, i.e., vs. patients with CASSC 0, 1, and 2), lower values of TC than patients with CASSC 0 and 1 (*p* < 0.05), lower values of LDL than patients with CASSC 1 (*p* < 0.05), and higher values of monocytes than patients with CASSC 0 (*p* < 0.05). There were no significant associations between SII or SIRI and severity of CAD (analysis of variance with four levels in Table 2); however, the highest values were observed for patients with CASSC 3. The highest values for patients with CASSC 3 were also presented in analysis performed without patients with CASSC 0 (analysis of variance with three levels in Figure 1). There was a significant result in the analysis of variance for SIRI, without significant differences in multiple comparisons. There were no significant differences in SII and SIRI between patients with CASSC 0 and CASSC 1–3 (Figure 2).

### 2.3. Association between Measured Parameters and Diagnosis

**Figure 3:**
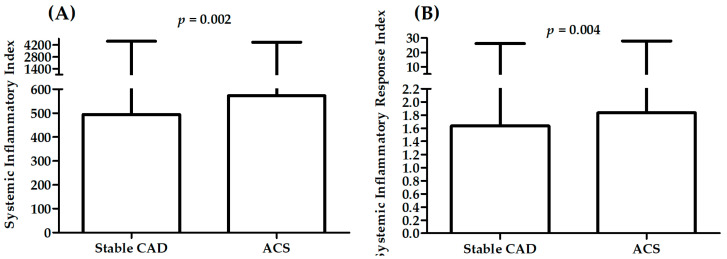
Differences in SII (**A**) and SIRI (**B**) between patients with stable CAD and ACS.

**Figure 4:**
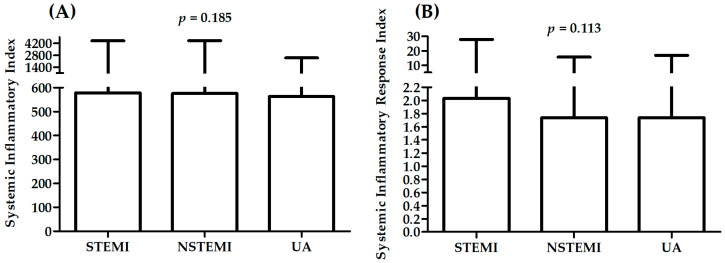
Differences in SII (**A**) and SIRI (**B**) between patients with STEMI, NSTEMI and UA.

**Figure 5:**
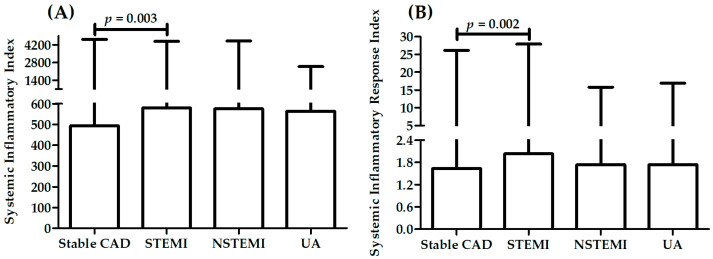
Differences in SII (**A**) and SIRI (**B**) between patients with different diagnosis.

Table 3 presents a comparison between patients with different diagnosis: stable CAD, STEMI, NSTEMI, UA in measured parameters. A significant difference in sex distribution was observed between patients with different diagnosis. There was also a significant difference in distribution of patients with history of previous MI, hyperlipidemia, hypertension, and smoking status. Patients with STEMI were younger than patients with other diagnoses (*p* < 0.001 vs. patients with stable CAD, and UA) and have the highest value of TC (*p* < 0.05 vs. patients with UA), LDL (*p* < 0.01 vs. patients with stable CAD and UA), leukocytes, neutrophils (*p* < 0.001 vs. patients with stable CAD), and monocytes (*p* < 0.05 vs. patients with stable CAD). Figure 3, Figure 4 and Figure 5 present results for SII and SIRI. There were significant differences in both SII and SIRI between patients with stable CAD and ACS (Figure 3): patients with ACS presented significantly higher values. However, there were no significant differences between patients with STEMI, NSTEMI, and UA (Figure 4). The highest values for patients with STEMI were presented in analysis of variance with four levels (Table 3, two last rows), significantly higher than in patients with stable CAD (Table 3, last rows, Figure 5).

### 2.4. Association of Sii and Siri with Selected Parameters

**Figure 6:**
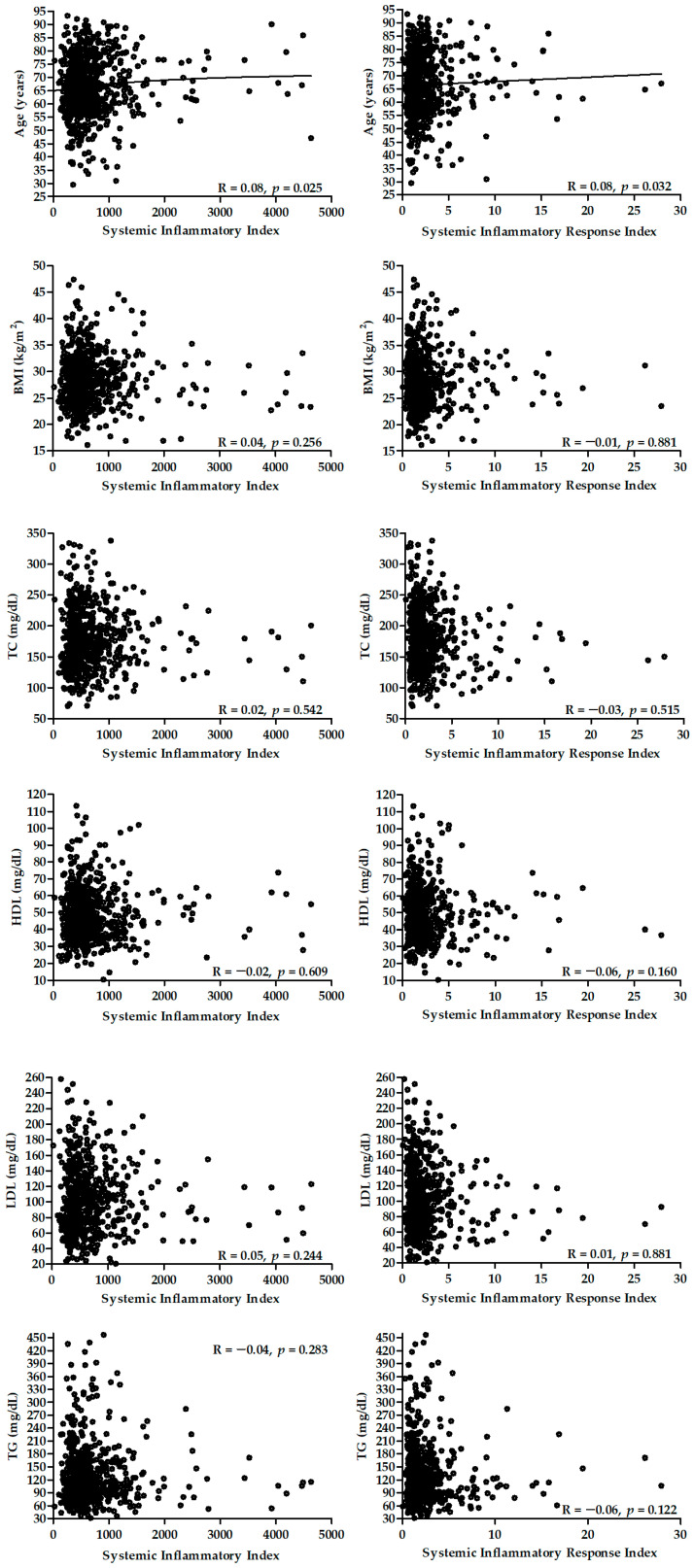
Correlation between SII (**left** panel) and SIRI (**right** panel) and age, BMI, and lipid profile.

Figure 6 presents the correlation between the SII and SIRI biomarkers and age, BMI, and lipid profile. There was a significant correlation between age and both biomarkers.

**Figure 7:**
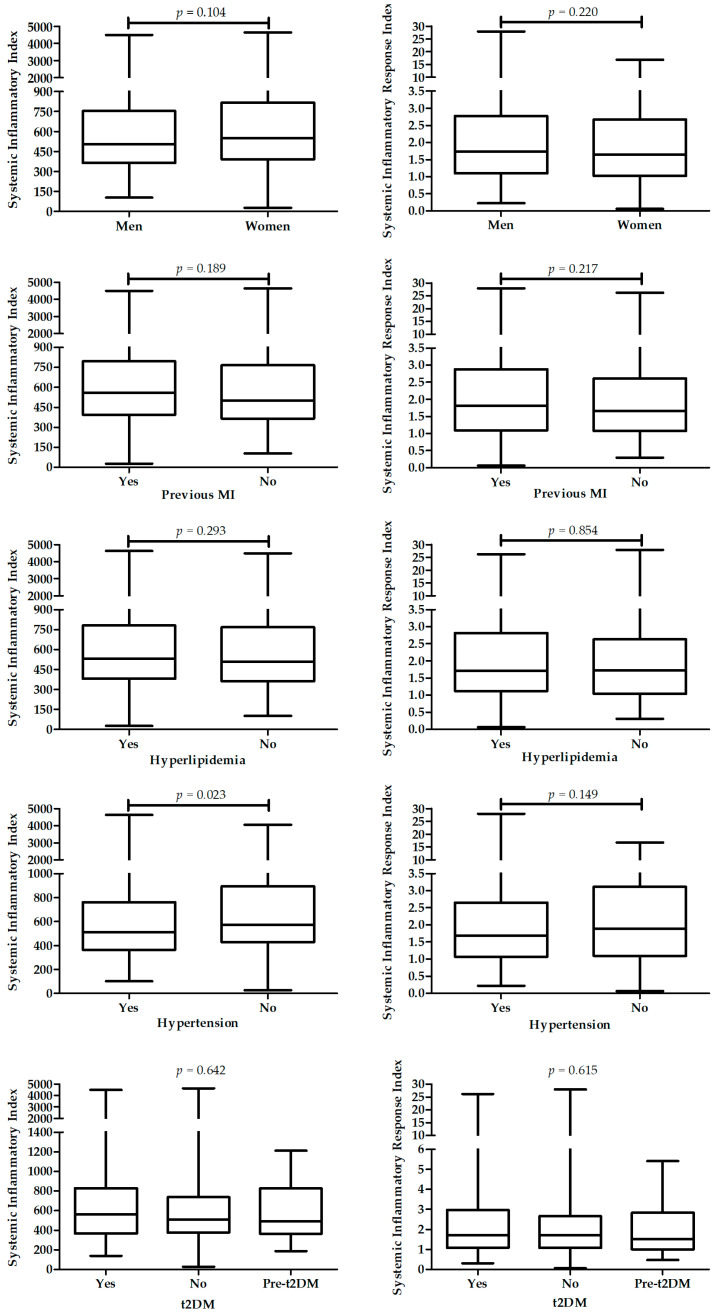
Differences in SII (**left** panel) and SIRI (**right** panel) between patients with different diagnosis.

Figure 7 presents differences in SII and SIRI between patients with different diagnosis.

## 3. Discussion

This study evaluated the relationship of two novel systemic inflammatory biomarkers, SII and SIRI, with the severity of CAD and the diagnosis of stable CAD versus ACS as part of a research project aimed at assessing the relationship of blood cell count as an inflammatory marker with CAD and its complications. We found that SII and SIRI were higher in patients diagnosed with STEMI, NSTEMI and UA compared to those with stable CAD; significantly higher values of these biomarkers were observed for patients with STEMI than those with stable CAD. Notably, the highest values of SII and SIRI were observed for patients with the highest stage of CAD, i.e., in patients with CASSC 3 in comparison to those with CASSC 0–2. In our previous study, we described significantly higher NLR values in patients undergoing coronary angiography due to subsequent ACS with a history of previous ACS compared to patients with stable CAD [44], as well as a lack of significant differences in platelet activity parameters (MPV and P-LCR) between these two groups [45]. In addition, the group of patients with three-vessel CAD had the highest NLR, but without statistical significance [44].

The immune system and inflammatory processes play a key role in the pathogenesis of atherosclerosis [46]. In patients with CAD, the elevation of standard inflammatory markers, e.g., white blood cell count (WBC) or CRP, was not only observed [47,48], but also associated with higher cardiovascular risk [49], severity of CAD, heart muscle perfusion [50,51], atherosclerotic plaque instability [52], and mortality due to CAD [53]. Chronic, low-degree inflammation seems to be crucial in the progression of CAD [54]. In ACS, inflammation is responsible for ischemic–reperfusion injury to the heart muscle [3,55]; thus, the benefits of lowering of the residual inflammatory risk with various treatments are being thoroughly researched [56,57,58,59]. However, the mechanisms of inflammation in ACS are not clearly described yet. Depending on pathogenesis, ACS was categorized into two groups: with and without systemic inflammation [3,60]. The available data do not recognize any significant association of plaque destabilization with systemic inflammation but indicate that various immune cells and pro-inflammatory cytokines contribute to this process [3]. Leucocytes and their subtypes (neutrophils, lymphocytes, and monocytes) are measured in total blood cell count, which is an affordable and accessible way to assess inflammatory processes that are involved in CAD pathogenesis [61] as well as modify ACS and stroke risk [11]. In addition, elevated WBC in patients with MI is an independent predictor of mortality [62]. Monocytes, a specific leucocyte subgroup, initiate and promote the progression of atherosclerosis by releasing pro-inflammatory cytokines, reactive oxygen species (ROS), and proteolytic enzymes [63]. Adhering to the endothelium, they differentiate into macrophages and subsequently, by absorbing lipids, they morph into foam cells, which activate cytokines and ROS release [64]. Monocyte count was described as a CVD mortality predictor, independently of other classic risk factors in long- [65] and short-term observations [66]. Neutrophils, the most abundant leukocyte subtype, exacerbate vessel wall inflammation by causing small muscle cell apoptosis [67,68]. Furthermore, a high neutrophil count correlates positively with the risk of plaque rupture [68] and increases the risk of thrombosis in the microcirculation [69]. However, lymphocytes hinder the progress of atherosclerosis [70]. Lymphopenia is positively correlated with MACE [71] and frequency of heart failure [72], as well as a poor prognosis in ACS patients [73]. Platelets have a twofold effect on atherosclerosis: their adhesion to the vessel wall promotes plaque formation [74], and their activation promotes inflammation and thrombosis [75,76].

To our knowledge, this study was the first to correlate elevated SIRI with CAD extension. The highest SIRI values were observed in three-vessel CAD (CASSC 3). However, SII did not correlate with the severity of CAD. So far, only a few papers have described the relation of SII with the extension of CAD. Liu et al. correlated SII with the Gensini scale of CAD severity in 400 patients who underwent coronary angiography. They described SII as an independent factor of the diagnosis and severity of CAD [25]. Similarly, Candemir et al. demonstrated a positive relationship between SII and CAD severity assessed with the SYNTAX scale in a group of 669 patients with stable CAD [26]. Erdogan et al. found that SII is positively correlated with the probability of finding a functionally significant stenosis in the coronary artery using fractional flow reserve [24].

One of the most important differences between SIRI and SII is that SIRI uses monocyte count and, on the contrary, SII employs thrombocytes. This may contribute to a substantial difference in the obtained results, as monocytes are the main cells responsible for the formation of atherosclerotic plaque. The lack of statistically significant difference between SII values observed in groups of patients with varying CASSC presented in this study could be the result of specific characteristics: nearly half of the cohort had ACS diagnosed shortly before the data were obtained, and more than 60% of them had another MI in the past. The discrepancies between our results and others referenced here may also be caused by different types of scales used to assess the severity of CAD.

Significantly higher SII and SIRI values were observed among patients with ACS (STEMI) compared to those with stable CAD in our cohort. The correlation of inflammatory biomarkers with ACS episodes was suggested previously [29,30]. The observation of more than 5000 patients with CAD treated with PCI showed that SII predicts the occurrence of MACE (ACS, stroke not resulting in death or death from heart disease) compared to classic risk factors [30]. These results were corroborated by Li et al., who not only found the relationship of lymphocyte-based inflammatory markers with MACE, but also reported the advantage of SIRI compared to other inflammatory markers in this setting [29]. Another 10-year observation of 85 thousand respondents revealed the correlation of elevated SIRI with higher frequency of ACS in patients under 60 years of age. However, the correlation with SII was not found [41].

The limitations of our study include the narrow research cohort in terms of number, relatively wide exclusion criteria (active neoplastic processes or paraneoplastic syndromes, diagnosed active viral or bacterial infection, chronic kidney disease (stages III–V), elevated erythrocyte sedimentation rate or serum CRP concentration), as well as poor socio-geographical diversity. The retrospective, cross-sectional, and observational character of this study disables the causal analysis of variables, as well as the confidence on the low-grade inflammation being due to the heart disease. The assessment of the severity of CAD was based on coronary angiography and the CASSC without the ability to address the stabilizing impact of coronary calcifications. The impact of hypolipidemic drugs could not be taken into account despite their possible impact on low-grade inflammation due to all patients being treated with comparable dose of statins.

The novel inflammatory biomarkers SII and SIRI require more in-depth and well-designed research on large groups of patients, as they may be a promising clinical tool for assessing CAD and its possible complications.

## 4. Materials and Methods

### 4.1. Study Population

A total of 699 patients (256 women) who underwent diagnostic coronary angiography due to CAD between 2013 and 2017 in the Cardiology Department of Bielanski Hospital, Warsaw, Poland, and agreed to participate in the study in writing, were included in this analysis. Every patient in this study was treated with HMG–CoA reductase inhibitor (atorvastatin or rosuvastatin) and acetylsalicylic acid. The study exclusion criteria were as follows: active neoplastic processes or paraneoplastic syndromes, diagnosed active viral or bacterial infection, chronic kidney disease (stages III–V), elevated erythrocyte sedimentation rate or serum CRP concentration.

### 4.2. Clinical and Laboratory Data

Demographic, anthropometric, and laboratory data were retrieved from patient electronic files. Blood samples for laboratory tests were collected with cephalic venipuncture and then examined in the hospital laboratory using standard clinical chemical analysis. Total blood count was measured in blood samples collected in di/tripotassium EDTA tubes using an automatic blood counter within two hours after venipuncture. CRP was measured using commercial laboratory assay used conventionally at the hospital laboratory. SII was defined as (neutrophil count) × (platelet count)/(lymphocyte count). SIRI was calculated as (neutrophil count) × (monocyte count)/(lymphocyte count). Obesity or overweight was diagnosed according to the WHO criteria [77]. Hypertension was defined as blood pressure exceeding 140/90 mmHg during office measurement, as described in the 2021 European Society of Hypertension Practice Guidelines [78]. The 2019 ESC Guidelines on diabetes, pre-diabetes, and cardiovascular diseases criteria were used to determine type 2 diabetes mellitus, which are as follows: fasting blood glucose levels exceeding ≥7.0 mmol/L (126 mg/dL) or hyperglycemia symptoms (including frequent urination, increased thirst, fatigue, acetone breath, nausea) accompanied by random blood glucose levels ≥11.1 mmol/L (200 mg/dL) or blood glucose at 120 min during an oral glucose tolerance test ≥11.1 mmol/L (200 mg/dL) [79]. According to the 2019 ESC/EAS Guidelines for the management of dyslipidemias, dyslipidemia was diagnosed in patients whose lipid profile did not meet the treatment goals for their respective risk level [80]. Coronary angiography was performed using radial or femoral artery access. As outlined in the 2021 ACC/AHA/SCAI Guideline for Coronary Artery Revascularization, this is the preferred method to assess stenosis in CAD [81]. Each coronary angiography was evaluated by three independent cardiologists. The results were quantified using the Coronary Artery Surgery Study Class (CASSC), which assigns one point for stenosis greater than 70% in a major coronary artery (left anterior descending artery, left circumflex artery, right coronary artery) and two points for left main coronary artery stenosis greater than 50%. The fractional flow reserve was used to assess cases where the degree of stenosis was unequivocal. The sum of points ranging from 0 to 3, reflecting, respectively, one-, two-, or three-vessel CAD, was entered into the database. The diagnosis of ACS was based on the criteria of the European Society of Cardiology guidelines, which are as follows: increased concentration of markers of myocardial injury with the coexistence of at least one of the specified below: symptoms of stenocardia, changes in the ECG suggesting ischemia, results of imaging tests depicting myocardial necrosis, or coronary artery thrombus identification on coronary angiography [82].

### 4.3. Statistical Analysis

The data distribution was determined using a Shapiro–Wilk test. Differences between prevalence in selected groups were determined with a Pearson’s chi-squared test or Fisher’s exact test. The parameters were compared between patients with different CASSC or between patients with different diagnosis using Kruskal–Wallis analysis of variance with multiple comparisons performed using Dunn’s test in case of significant differences. Mann–Whitney U test was used to compare the results between the two groups. The relationship between the selected variables was analyzed with a Spearman correlation coefficient (R). Statistical significance was recognized if a two-sided *p*-value < 0.05. Statistical analysis was completed with Statistica 13 (StatSoft Inc., Tulsa, OK, USA). Figures were drawn with GraphPad Prism 8.0 (GraphPad Software, San Diego, CA, USA).

## 5. Conclusions

ACS patients (STEMI, NSTEMI, UA) had elevated values of the novel inflammatory markers SII and SIRI compared to those with stable CAD. SIRI and SII reached the highest values for patients with three-vessel CAD. The relationship between the inflammatory parameters of SII, SIRI and their components and the factors that influence the pathogenesis of CAD need further research, as does the role of the mentioned markers in the prediction of ACS.

## Figures and Tables

**Table 1:** Characteristics of participants.

Variable	Values
N of participants [♂/♀]	699 (444/256)
Age [years]	66.3 (29.5–93.3)
BMI [kg/m^2^]	27.8 (16.1–47.4)
Cause of hospitalization [stable CAD/STEMI/NSTEMI/UA]	366/147/108/78
Previous MI [yes/no]	269/430
Total cholesterol (TC) [mg/dL]	172.0 (70.3–338.3)
High-density lipoprotein (HDL) [mg/dL]	47.2 (10.4–113.2)
Low-density lipoprotein (LDL) [mg/dL]	95.7 (20.5–257.9)
Triglycerides (TG) [mg/dL]	113.9 (31.3–456.7)
Hyperlipidemia [yes/no] (N = 644)	377/267
Hypertension [yes/no]	577/122
Smoking [active/former smoker/no]	195/75/429
Type 2 diabetes mellitus (t2DM) [yes/pre-diabetes/no]	236/30/433
Coronary Artery Surgery Study Class (CASSC) [0/1/2/3]	177/193/174/155
Leukocytes [thousand cells/µL] (N = 694)	8.0 (3.0–26.0)
Platelet (PLT) [mcL]	220 (150–445)
Neutrophils [thousand cells/µL]	4.8 (1.4–44.7)
Monocytes [thousand cells/µL]	0.7 (0.2–3.0)
Lymphocytes [thousand cells/µL]	1.9 (0.4–41.8)
SII	519 (26–4634)
SIRI	1.7 (0.06–27.9)

**Table 2:** Association between selected parameters and CAD stages.

Variable	CASSC 0	CASSC 1	CASSC 2	CASSC 3	*p*-Value
N of participants [♂/♀]	85/92	124/69	127/47	107/48	<0.001
Age [years]	65.9 (36.7–91.5)	65.2 (34.7–93.3)	64.0 (33.6–89.2)	68.9 (29.4–90.8)	0.019
BMI [kg/m^2^]	27.8 (17.3–47.4)	27.1 (16.9–43.4)	27.8 (17.4–44.6)	28.3 (16.1–45.9)	0.635
Cause of hospitalization [stable CAD/STEMI/NSTEMI/UA]	147/8/12/10	71/63/36/23	81/39/30/24	67/37/30/21	<0.001
Previous MI [yes/no]	13/164	79/114	84/90	93/62	<0.001
TC [mg/dL]	179.4 (73.3–328.7)	178.9 (70.3–331.7)	165.5 (70.9–338.3)	161.7 (84.4–310.8)	0.007
HDL [mg/dL]	53.8 (10.4–107.6)	46.6 (20.5–97.4)	46.7 (21.3–113.2)	44.3 (14.6–89.2)	<0.001
LDL [mg/dL]	95.8 (20.5–230.7)	105.0 (23.5–251.7)	94.0 (24.4–258.0)	87.0 (22.3–228.3)	0.048
TG [mg/dL]	115.3 (31.3–340.0)	110.6 (43.6–438.3)	113.4 (38.0–456.7)	115.4 (47.6–391.8)	0.847
Hyperlipidemia [yes/no]	89/71	115/61	92/70	81/65	0.197
Hypertension [yes/no]	132/45	157/36	152/22	136/19	0.003
Smoking [active/former smoker/no]	30/13/134	69/17/107	57/24/93	39/21/95	<0.001
t2DM [yes/pre-diabetes/no]	51/7/119	57/6/130	62/10/102	66/7/82	0.070
Leukocytes [thousand cells/µL]	7.7 (3.9–21.2)	8.1 (3.9–26.0)	8.0 (3.6–19.6)	8.2 (3.0–18.1)	0.085
PLT [mcL]	212 (150–445)	224 (150–438)	223 (151–439)	218 (150–429)	0.570
Neutrophils [thousand cells/µL]	4.6 (1.7–44.7)	4.8 (1.7–23.8)	4.8 (1.4–16.8)	5.0 (1.4–14.2)	0.260
Monocytes [thousand cells/µL]	0.7 (0.3–1.7)	0.7 (0.3–2.4)	0.7 (0.3–2.0)	0.8 (0.2–3.0)	0.046
Lymphocytes [thousand cells/µL]	1.9 (0.4–4.6)	2.1 (0.4–4.9)	2.0 (0.7–41.8)	1.9 (0.6–5.0)	0.162
SII	505 (103–4932)	510 (142–4467)	507 (26–2574)	566 (136–4191)	0.277
SIRI	1.6 (0.3–16.7)	1.7 (0.3–27.9)	1.7 (0.1–19.4)	1.9 (0.5–15.2)	0.066

**Table 3:** Differences in selected parameters between patients with different diagnosis.

Variable	Stable CAD	STEMI	NSTEMI	UA	*p*-Value
Number of participants [♂/♀]	225/141	110/37	67/41	41/37	0.005
Age [years]	67.1 (29.4–93.3)	63.0 (36.3–89.1)	65.1 (36.1–92.1)	69.6 (33.6–91.5)	<0.001
BMI [kg/m^2^]	28.0 (16.1–47.4)	26.9 (16.9–44.6)	27.7 (17.8–42.4)	28.6 (17.3–43.4)	0.549
Previous MI [yes/no]	109/257	71/76	63/45	26/52	<0.001
TC [mg/dL]	170.4 (84.8–328.7)	180.6 (97.4–320.3)	168.9 (70.3–338.3)	159.2 (81.4–331.7)	0.026
HDL [mg/dL]	50.2 (10.4–113.2)	45.0 (20.5–92.9)	42.6 (22.8–78.9)	46.5 (19.5–72.9)	<0.001
LDL [mg/dL]	89.0 (20.5–258.0)	108.2 (28.3–214.1)	100.1 (23.5–244.3)	86.8 (32.9–251.7)	<0.001
TG [mg/dL]	115.8 (35.7–438.3)	107.6 (47.6–367.8)	111.2 (43.6–456.7)	114.7 (31.3–251.7)	0.971
Hyperlipidemia [yes/no]	187/151	99/44	59/38	32/34	0.011
Hypertension [yes/no]	289/77	119/28	93/15	76/2	<0.001
Smoking [active/former smoker/no]	70/56/240	67/8/72	45/3/60	13/8/57	<0.001
t2DM [yes/pre-diabetes/no]	127/16/223	44/3/100	33/8/67	32/3/43	0.236
Leukocytes [thousand cells/µL]	7.8 (3.6–18.6)	8.7 (3.0–26.0)	7.9 (3.9–21.2)	7.8 (4.7–19.6)	0.0006
PLT [mcL]	214 (150–445)	230 (150–428)	224 (151–410)	219 (150–432)	0.099
Neutrophils [thousand cells/µL]	4.7 (1.4–44.7)	5.3 (1.4–23.8)	4.8 (1.7–19.3)	4.8 (2.3–15.1)	0.0008
Monocytes [thousand cells/µL]	0.7 (0.2–1.8)	0.8 (0.3–2.0)	0.7 (0.3–3.0)	0.7 (0.3–2.4)	0.030
Lymphocytes [thousand cells/µL]	1.9 (0.6–41.8)	2.0 (0.6–5.3)	1.9 (0.4–3.8)	2.0 (0.4–4.6)	0.756
SII	494 (26–4634)	579 (142–4467)	576 (103–4491)	563 (192–2482)	0.0052
SIRI	1.6 (0.1–26.2)	2.0 (0.6–27.9)	1.7 (0.3–15.8)	1.7 (0.5–16.9)	0.0053

## Data Availability

Data can be provided by the corresponding author upon reasonable request.
